# Clinical Feasibility of Speech Phenotyping for Remote Assessment of Neurodegenerative and Psychiatric Disorders (RHAPSODY): a study protocol

**DOI:** 10.1192/j.eurpsy.2022.435

**Published:** 2022-09-01

**Authors:** R. Taylor, E. Hampsey, M. Mészáros, C. Skirrow, R. Strawbridge, L. Chok, D. Aarsland, A. Al-Chalabi, K.R. Chaudhuri, J. Weston, E. Fristed, A. Young, O. Awogbemila

**Affiliations:** 1 King’s College London, Institute Of Psychiatry, Psychology, & Neuroscience, London, United Kingdom; 2 Novoic Limited, Novoic Limited, London, United Kingdom; 3 Kings College Hospital and Kings College, Parkinson Foundation International Centre Of Excellence, London, United Kingdom

**Keywords:** Speech, affective disorders, Neurodegenerative Disorders, Artificial intelligence

## Abstract

**Introduction:**

The diagnosis of neurodegenerative and psychiatric disorders (NPDs) in primary care can suffer from inefficiencies resulting in misdiagnoses and delayed diagnosis, limiting effective treatment options. The development of speech and language-based profiling biomarkers could aid in achieving earlier motor diagnosis for PD for instance, or more accurate diagnosis of clinically similar or late presenting NPDs.

**Objectives:**

RHAPSODY aims to investigate the feasibility of the remote administration of a battery of speech tasks in eliciting continuous narrative speech across a range of NPDs. The project also aims to determine the feasibility of using acoustic and linguistic biomarkers from speech data to support the clinical assessment and disambiguation of common NPDs

**Methods:**

All participants (n=250) will take part in a single virtual telemedicine video conference with a researcher in which they are screened and complete a battery of speech tasks, in addition to cohort-specific screening measures. Over the following month, participants will be asked to complete a series of short, self-administered speech assessments via a smartphone application.

**Results:**

The speech tasks will be audio-recorded and analysed on Novoic’s technology platform. Objectives will be analysed using measures including average length of speech elicitation for speech tasks, intra- and inter-subject variance, differences in linguistic patterns, and response rates to speech assessments.

**Conclusions:**

The analyses could help to identify and validate speech-derived clinical biomarkers to support clinicians in detecting and disambiguating between NPDs with heterogeneous presentations. This should further support earlier intervention, improved treatment options and improved quality of life.

**Disclosure:**

In terms of significant financial interest and relationships, it is emphasised that the private organisation Novoic, who aim to develop speech algorithms for diagnostic use, is the study’s sponsor and employees or former employees of this company comprise

